# Recurrent summer drought temporarily stimulates fine root growth but enhances winter root losses in alpine grassland

**DOI:** 10.3389/fpls.2025.1625076

**Published:** 2025-07-30

**Authors:** Patrick Möhl, Erika Hiltbrunner

**Affiliations:** ^1^ Physiological Plant Ecology, Department of Environmental Sciences, University of Basel, Basel, Switzerland; ^2^ Centre for Sustainable Soils, Lancaster Environment Centre, University of Lancaster, Lancaster, United Kingdom

**Keywords:** climate change, leaf length, machine learning, neural network, rhizotron, root phenology, winter, snowmelt

## Abstract

By the end of the 21^st^ century, frequent droughts and substantial shifts in snowmelt are expected to massively impact the biomass production of alpine grasslands. While the biomass of alpine plants consists to >80% of roots, little is known about the root growth dynamics in these ecosystems. To fill this gap, we capitalized on a longer-term experiment in the Swiss Alps with annually recurring treatments imposing advanced and delayed snowmelt and summer drought lasting 5 and 10 weeks. Over 3–4 growing seasons (2019–2022), we weekly quantified total root length of the plant community at two different soil depths (0–10 cm and 10–20 cm) using 90 minirhizotrons in 45 plots. We jointly assessed leaf elongation (of six abundant plant species) as proxies for the dynamics of biomass production. Increases in root length during summer continued beyond canopy development, with the duration of net root growth roughly double that of leaf expansion. Earlier and later snowmelt did not affect the proxies for total growth of leaves or roots but simply shifted their growing phases. Drought reduced leaf elongation across plant species whereas root length was stimulated by the 5-wk (not the 10-wk) drought in two seasons (+19% on average, 2020–2021). Natural rewetting after drought increased root growth by 38–77% compared to controls, but only in the 2020 growing season. Total root length in the topsoil declined by 7–15% during the last two winters, amounting to about one fourth of the previous seasons’ increase in root length. These root losses were 1.5 times higher following the 10-wk drought treatment. Our results highlight that earlier snowmelt alone will not stimulate productivity in alpine grassland. Root growth responses to drought depend on its duration and the long winter periods contribute to root losses, particularly in combination with severe drought in the preceding growing season.

## Introduction

The timing of plant development and growth is of fundamental importance for the ecosystem-wide uptake and cycling of resources (Nord and Lynch, 2009) but is also key to biotic interactions (Yang and Rudolf, 2010). Growth dynamics are strongly affected by climate change in many ecosystems, as a warmer spring and autumn lead to an advance and extension of seasonal growth in temperate regions (Jeong et al., 2011). Moreover, the timing of growth is a crucial determinant of plant responses to climate change (Richardson et al., 2018). For instance, drought may have the strongest effects on the seasonal biomass production when coinciding with peak growth (D’Orangeville et al., 2018; Lemoine et al., 2018). Drought may also delay growth until rewetting (Xu et al., 2010), providing leeway for compensatory growth (Hahn et al., 2020). Therefore, seasonal plant growth dynamics and its variability in a changing environment are central for assessing the consequences of climate change for biomass production. Although the dynamics of aboveground plant biomass have received considerable attention in grasslands—using practical proxies such as the normalized difference vegetation index (NDVI; Badeck et al., 2004; Dronova and Taddeo, 2022)—the same processes are notoriously understudied in roots. Our perception of seasonal growth dynamics thus largely reflects aboveground processes (Blume-Werry, 2022; Liu et al., 2022; Radville et al., 2016), while relatively little is known about the seasonal growth dynamics of roots, how these dynamics are linked to those of leaves, and, in particular, how they respond to climate change.

Disregarding the dynamics of root growth is especially problematic in high elevation grasslands, where most plant biomass is belowground (up to 90%, Körner, 2021). These ecosystems are also considered to be strongly affected by climate change (Winkler et al., 2019) because they are adapted to cold temperatures while experiencing above-average climate warming (Pepin et al., 2022). In temperate alpine vegetation with a strongly seasonal climate and short growing seasons of 6–12 weeks (Körner, 2021), higher spring temperatures due to climate warming lead to earlier snowmelt (Marty et al., 2023; Vorkauf et al., 2021b), substantially prolonging the timeframe for plant growth. Additionally, drought is becoming more frequent at high elevation even in places such as the Alps (Gobiet et al., 2014; Kotlarski et al., 2023), where water availability during the snow-free period is usually high. Earlier snowmelt and long periods without rain may lead to shifts in seasonal plant growth and impact the annual biomass production both above and below the ground.

In temperate alpine grasslands, leaf growth of most species starts a few days after snowmelt (Choler, 2015), even in years with extremely early snowmelt (Vitasse et al., 2017). A recent study with excavated monoliths of alpine grassland showed that growth of shoots and roots can be initiated months before natural snowmelt, at least with environmental conditions that mirror alpine summer (Möhl et al., 2022), suggesting that growth initiation is highly opportunistic in spring. However, this earlier start of the season led to earlier senescence and cessation of root growth, explaining why the annual biomass production is largely independent of snowmelt timing in this grassland (Möhl et al., 2023). Our understanding of the interannual variability in root growth *in situ* is still greatly limited by a lack of studies that cover more than a few timepoints per season (Mähr and Grabherr, 1983), while the response of inter- and intra-seasonal root growth dynamics to extreme events such as drought is entirely unknown in alpine grassland.

Drought has pronounced effects on soils and plants, commonly reducing soil nutrient mobility and their uptake by plants as well as leaf turgor pressure and transpiration. In most ecosystems and also in alpine grasslands, droughts can substantially reduce biomass production aboveground (Gilgen and Buchmann, 2009; De Boeck et al., 2015), whereas root growth is often maintained or even stimulated under moderate drought (Guasconi et al., 2023; Liu et al., 2018; Möhl et al., 2023) to enhance water and nutrient uptake (Chaves et al., 2003; Comas et al., 2013). Drought may also shift root growth to deeper soil layers that remain moist for longer (Zheng et al., 2024), or plant species with deep roots may cope better with extended dry periods (Künzi et al., 2025). In various grassland ecosystems, the effects of drought on plant growth were found to depend on drought timing and duration (Denton et al., 2017; Li et al., 2022; Zeiter et al., 2016; Zhang et al., 2020), likely shaped by the temporal growth dynamics within the growing season. In addition to the direct effects of drying soil on plant growth, post-drought rewetting and its associated nutrient bursts (Barnard et al., 2020; Schimel, 2018) play a decisive role during recovery (Hahn et al., 2020; Schärer et al., 2023).

The long winters of seasonal alpine and arctic environments commonly remain unaccounted for in studies on biomass production, as snow-cover or extremely low temperatures may prevent growth over winter. Yet, even minute growth rates allow alpine specialists, such as the snowbed species *Soldanella pusilla*, to form a complete inflorescence under snow over the long winter period (Körner et al., 2019). A continuous snow layer insulates the ground, resulting in a constant temperature of 0 °C in the topsoil, where the majority of roots are (Iversen et al., 2015; Körner, 2021). Roots of alpine plants are capable of growing at 1–2 °C (Nagelmüller et al., 2017), and since the reduction in growth rate at critically low temperature is an asymptotic process, we speculate that root growth over winter, despite being slow, may contribute to the annual root production. Winter root growth was evidenced in many lower elevation ecosystems (Radville et al., 2016), but little is known about how root growth in alpine ecosystems differs between summer and winter.

That we know comparably little about root growth dynamics stems from the difficulties associated with *in situ* root sampling: Excavating and quantifying roots from intact soil is very laborious and subtle changes in root biomass over short intervals are prone to be lost in the background variation of the extremely large root biomass in alpine grassland (Mähr and Grabherr, 1983). The ingrowth-core method can partly overcome these problems but requires a minimal incubation time that is too long to capture short-term dynamics. Moreover, offering root-free soil may induce an overestimation in root production in ingrowth cores because root-free space represents a rather unusual condition for this densely rooted grassland. Rhizotrons (‘soil windows’) can overcome these limitations, but repeated measurements in experimental field studies quickly add up to a large number of images that are time-consuming to analyze manually. Recent advances in machine learning have led to the development of neural networks that recognize roots (Atkinson et al., 2019; Jiang and Li, 2020), which enable researchers to assess changes in root area and length at unprecedented resolution—spatially, temporally, and in terms of image resolution. Scanners with 1200 DPI and more are required to accurately depict roots in grassland ecosystems with dense networks of fine roots, which often have diameters of less than 0.5 mm (Möhl et al., 2022; Song et al., 2024; Wu et al., 2014). The resulting sequences of ‘standing’ root lengths are highly valuable as they reflect the dynamics of the plants’ current capacity for water and nutrient uptake. In addition, these sequences can serve as an indicator of growth in ecosystems where root turnover is slow, such as the alpine grassland studied here (>6 years fine root longevity; Schäppi and Körner, 1996; Budge et al., 2011)—provided the sequences are in high temporal resolution.

In this study, we investigate *in situ* the temporal dynamics of leaf and root expansion (referred to as leaf and root growth in the following) over the four years 2019–2022 of a longer climate change experiment in an alpine grassland, which aims to assess the individual and combined effects of altered snowmelt timing (advanced and delayed) and summer drought. Measured weekly during the ‘meteorological growing season’ (*sensu* Körner et al., 2023), we combine simple leaf lengths measures for the six most abundant species with community-level root growth derived from 90 minirhizotrons, analyzed at two different soil depths. We aim at testing the following hypotheses: H1: Temporal shifts in leaf elongation under altered snowmelt timing are paralleled by changes in root length. H2: Drought forces roots to deeper soil, while rewetting stimulates root elongation across soil depths. H3: Root length increases during winter but the produced amount is negligible.

By collecting above and belowground data at high temporal resolution over multiple growing seasons, we offer insights into the responses of alpine grassland to climate change and provide a baseline for future studies that examine the effects of altered life conditions and disturbances on the biomass production and its temporal dynamics in similar semi-natural ecosystems.

## Materials and methods

### Study site

The experimental site was located in the Swiss Alps at an elevation of 2480 m (46° 33′ 47′′ N, 8° 23′ 28′′ E) on late-successional alpine grassland dominated by the sedge *Carex curvula*, typical for soils on acidic bedrock above the treeline in the Alps and other European mountains (Landolt, 2012; Puşcaş and Choler, 2012). Annual biomass production amounts to 120–160 g m^-2^, of which about 60% is accounted for by graminoids and 40% by forbs (Möhl et al., 2020). Beside *Carex curvula*, the most abundant species include the grasses *Helictotrichon versicolor* and *Anthoxanthum alpinum* and the forbs *Geum montanum*, *Leontodon helveticus* and *Potentilla aurea* (hereafter referred to by genus name). The soil is acidic, with a pH_CaCl2_ between 3.6 (0–10 cm) and 4.2 (10–20 cm), 60–100 cm deep and considered a partially podzolic cambisol. The permanent wilting point derived from replicated pF curves is 10.1 vol-% at 10 cm and 8.8 vol-% at 30 cm soil depth (Vorkauf et al., 2021a). Roots grow very densely in the topsoil (0–10 cm) but become scarce in deeper soils. Snowmelt usually occurs in June to early July, and the meteorological growing season lasts until late September, when freezing temperatures and snowfall become more frequent. Monthly precipitation during the growing season amounts to 50–150 mm. In winter, plants are usually covered by a snow layer of up to four meters and snow-covered soil remains unfrozen (constant at 0 °C).

### Experimental setup

**Figure 1:**
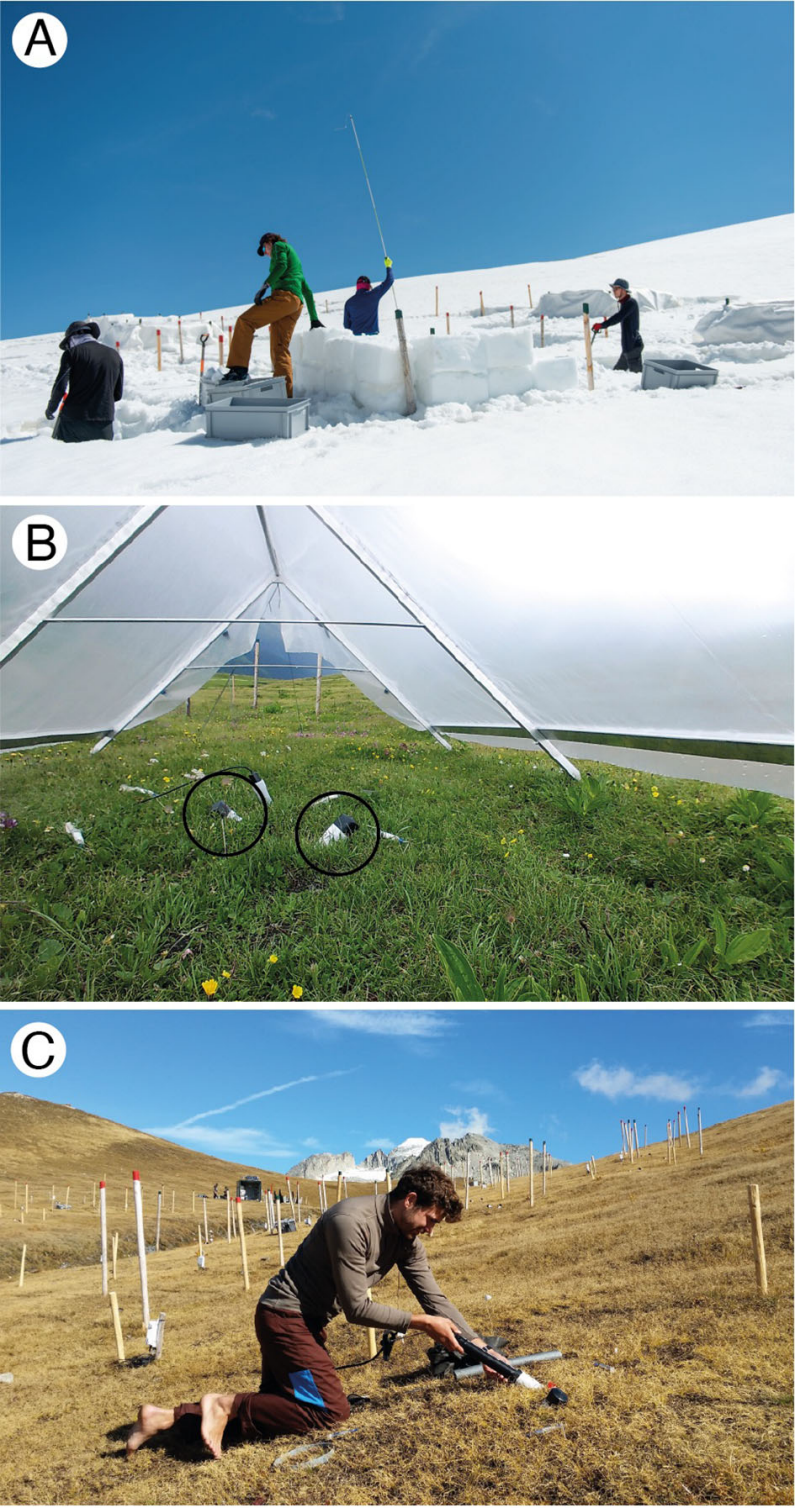
**(A)** Snow manipulations in spring prior to natural snowmelt; snow height of the plot in the front is being raised to 1.3 m and then covered by a white fleece in order to delay natural snowmelt. **(B)** Close-up of the vegetation and two rhizotron tubes (encircled) under a rain-out shelter. **(C)** Root scanning in autumn (1 Oct 2019). Wooden poles in the background mark plots for snow manipulations in the following spring.

We capitalized on a longer-term experiment that was established in 2016 to study the individual and combined effects of snowmelt timing and drought on alpine grassland (**Figure 1**), described in detail in Möhl et al. (2023). In brief, we established a 3x3 full-factorial experiment including three levels of manual snow manipulation (control at ca. 1 m snow depth, snow removal down to 0.5 m, snow addition to 1.3 m and then covered by white fleece) and three levels of rain exclusion with different duration (control, 5-week drought, 10-week drought). A total of 45 plots (2 x 2.5 m) were assigned to a snow manipulation and drought treatment (n = 5 for all combinations), grouped into five spatially separated blocks. Snow manipulations were conducted each spring from 2016 to 2022 (**Figure 1A**). Immediately after snowmelt, rain-shelters (2.5 x 3 m) were placed over 30 plots to simulate summer drought from 2017 to 2022 (**Figure 1B**). Shelters were removed five (5-wk drought, n = 15) and ten (10-wk drought, n = 15) weeks after placement.

### Environmental conditions

Precipitation, air temperature, relative humidity and solar radiation were monitored 1.5 m above ground by two on-site weather stations (Vantage Pro2, Davis Instruments Corp., US). Soil temperature was measured hourly at 3–4 cm depth, representative for plant meristems and close to the two rhizotron tubes in the center of each plot (TidbiT v2 Temp, Onset Computer Corp., US). Soil temperature readings were used to pinpoint the exact snowmelt timing and to calculate growing degree hours (GDH > 5 °C) throughout the season. Two soil temperature sensors failed in 2020, one in 2021, and one in 2022. In these cases, snowmelt timing was inferred from webcam images and GDH from a nearby plot of the same treatment (R^2^ = 0.99 prior to logger failure). Soil moisture was assessed weekly during the growing season throughout the duration of this study (2019–2022). Topsoil moisture (0–5 cm depth; ML3 volumetric soil moisture probe, Delta-T Devices, UK) was measured in (and averaged across) each corner and the center of the central m^2^ (1 x 1 m) of each plot, while moisture of deeper soil layers was measured in the center only (5–10, 15–20, 25–30 and 35–40 cm depth, Profile probe PR2, Delta-T Devices, UK).

### Leaf growth

In alpine grasslands with short growing seasons, most of the seasonal aboveground biomass is formed within the first weeks after snowmelt until peak biomass, followed by a very short phase of stable biomass that then transitions into senescence. Hence, leaf expansion from snowmelt to peak biomass reflects the major dynamic of leaf growth in alpine plant species. To assess how our experimental treatments affect leaf growth, we monitored leaf expansion of the six most abundant species during three growing seasons (2019–2021), namely of *Carex*, *Helictotrichon, Anthoxantum*, *Geum*, *Leontodon*, and *Potentilla* (ordered by abundance). Each week, from snowmelt to late summer, we measured the length of up to 12 leaves (depending on species abundance) among the longest leaves of each species spread out over the central m^2^ of each plot. The length was assessed from the soil surface to the tip of the leaf with a precision of 0.5 cm. Only plots with at least four individuals were considered for the analysis of each species. In *Carex*, only the length of the green part was measured while the dead leaf tips were excluded. Leaf lengths of these six species were significantly related to the seasonal peak biomass of each year (biomass harvest data from Möhl et al., 2023; P < 0.001, R^2^ = 0.74).

### Root growth

Between 10 and 19 July 2019, we installed two transparent, acrylic rhizotron tubes (length = 50 cm, outer ⌀ = 5.6 cm) in the central m^2^ per plot (90 tubes in total). Holes were drilled with a hand auger (⌀ = 7 cm; Edelmann auger, Eijkelkamp, NL) and a guiding stand with an angle of 45° (Freschet et al., 2021a). The effective angles between soil surface and tubes averaged at 42.7 ± 4.3° (± SD, ranging from 32–61°). To avoid scratching the tube surface during installation, a dummy tube was inserted into the drilled hole before filling the gap between tube and soil (7 mm) with homogeneous, root-free, sieved soil from the site. The dummy tube was then carefully pulled out and replaced with a new tube. On average, the root images reached a soil depth of 20.7 cm and covered a soil area of 540 cm^2^. The lower opening of the tube (in the soil) was permanently plugged, while the upper opening (outside the soil) was closed with a removable lid. The part of the tube outside the soil was wrapped with layers of black and white tape to block sunlight. Removable polyethylene foam insulated the tubes inside.

The tubes were scanned at weekly intervals during the 2019–2022 growing seasons (**Figure 1C**). Two identical root scanners (CID-602, CID BioScience, US) were used to take 360°-images (1200 DPI resolution) of the roots growing along the tube surface. Two scans were required for each tube to capture the entire belowground extent. First roots were visible already one week after installation of the rhizotrons. To relate changes in root length (see below) to species-specific plant cover aboveground, we visually assessed plant species composition of the central m^2^ by cover estimates (0–100%) of each plant species in August 2022. In addition, we estimated the cover of the nine most abundant species in the immediate environment (45 x 25 cm) of each rhizotron tube based on photographs taken around the same time (estimates in 5% cover steps, 1% for species presence without substantial cover).

### Root image analyses

**Figure 2:**
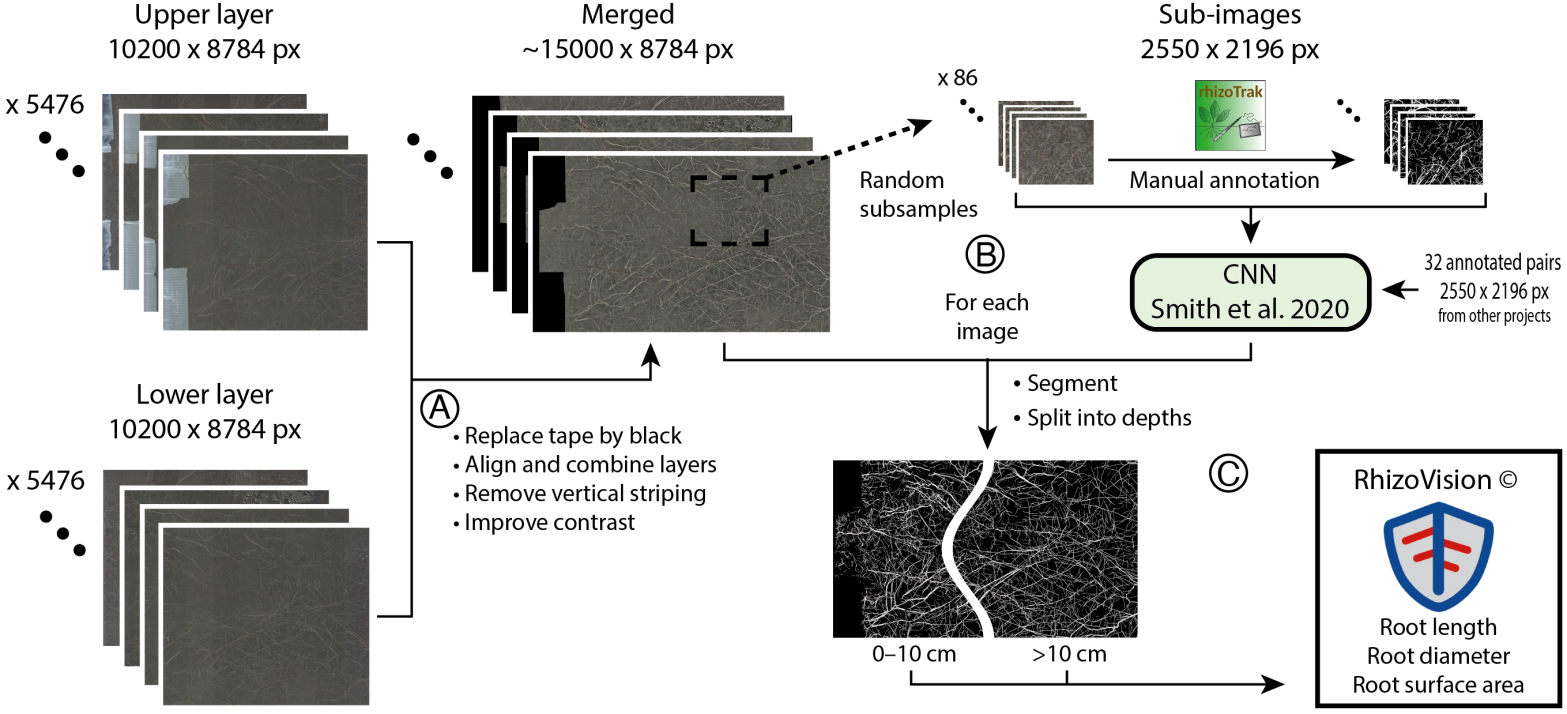
Image processing to acquire segmented images for two soil depths, starting with two images per rhizotron tube. **(A)** Several pre-processing steps were required to prepare the root images for automatic segmentation. **(B)** Sub-images were manually annotated (rhizoTrak plugin for Fiji). The resulting binary images (black = soil, white = roots) were used to train a neural network to generate binary images out of the pre-processed images. To improve learning, manually segmented images from a previous project were included in the training dataset. **(C)** All pre-processed images were converted into binary images using the neural network and then split into two depths (0–10 cm, 10–20 cm; by taking into account tube-specific parameters), before the relevant root measures were extracted using RhizoVision Explorer (v. 2.0.3).

In total, we obtained 10,456 images from 90 rhizotron tubes, two soil layers, and 64 dates across four growing seasons. In 40 instances, data for only one soil layer was acquired, because the other was either not scanned or the scan was faulty. Scans from three dates (03/09/2020, 17/08/2022, 08/09/2022) were omitted because of unusually low soil-root contrast, which led to issues with automatic root detection. Raw images were prepared for segmentation using Python (v. 3.6.9; **Figure 2**). First, the light-blocking tape, visible in the upper scan close to the soil surface, was replaced by black pixels based on a mask that was manually generated for each tube and then automatically aligned for all images per tube. The two scans for each tube and date were merged into one image, covering the entire belowground length of the tube (**Figure 2**). All images from the same tube were then aligned to the first image of the time-series (planar shifts determined by phase correlation). Striping artifacts were removed following Jiang and Li (2020) before contrast and brightness were normalized.

From these images, 86 sub-images (2550 x 2196 px) from a random selection of tubes, treatments and years were manually annotated using the rhizoTrak plugin for Fiji (Möller et al., 2019; Schindelin et al., 2012). We used 61 of these annotated images for training and 25 for validation of a convolutional neural network (Smith et al., 2020). To improve the model, the training dataset was augmented with 32 images from a previous project of the same vegetation type (Möhl et al., 2022). The model was trained for 50 epochs (cycles through the entire dataset). In the best-performing model, 83% of all pixels predicted as root actually belonged to roots and 83% (coincidentally same value) of the actual root pixels were identified as such in the validation images. This model was applied to all images and the resulting binary images (white pixels = root, black pixels = soil) were split into soil depths of 0–10 cm and 10–20 cm (on average to 21.7 cm depth, depending on tube installation depth and angle). To do so, we calculated the corresponding pixel positions of 10 cm soil depth for each tube with respect to the soil-tube angle (**Figure 2**). Root length (mm) and root surface area (mm^2^) were extracted using RhizoVision Explorer (v. 2.0.3; Seethepalli et al., 2020) for different diameters classes (0–1 mm in 0.1 mm steps and >1mm). To account for differences in tape width and installation depth as well as angle, we expressed the root data per unit rhizotron image area (cm^-2^). We here present root length only, which was highly correlated with root surface area (Pearson’s r = 0.98 across all measurements) and this correlation did not differ between treatments or soil depth. Root length (and area) derived from automatically segmented images correlated well with those from manually annotated validation images (R^2^ > 0.96, **Supplementary Figure S1**). Although we here quantified changes in net root length without differentiating between root growth and root losses (which would require the tracking of the fate of individual roots), we assume that the weekly net changes provide a reliable estimate of root growth. Weekly mortality rates are expected to be very low during our observation period, as the average root longevity in this grassland is considered longer than the total study duration (e.g., Schäppi and Körner, 1996). This fits with the observation that dead-looking roots were extremely rare in manually annotated images (<0.1% of total root area; data not shown).

### Specific root length

To assess possible treatments effects on the specific root length (SRL), fine roots from each of three ingrowth cores per plot (⌀ = 4.4 cm, depth = 0–10 and 10–20 cm, incubated from September 2019 to September 2021) were collected. These fine roots were washed, scanned with a flatbed scanner (Epson Perfection V700 Photo; Seiko Epson Corp., JN) and dried at 80 °C. Root length and average diameter were quantified using WinRhizo (Regent Instruments Inc., CA) and SRL (in m g^-1^) was calculated by dividing the length by the dry weight.

### Statistical analyses

All statistical analyses were performed with the statistical programming language R v. 4.2.2 (R Core Team, 2022). The start and end of the peak growing period were defined as the surpassing of 20% and 80% of seasonal leaf expansion or root growth (maximum value minus first value). To extract the date or temperature sums (GDH) when growth surpassed these thresholds, we fitted generalized additive models (GAMs; mgcv package, Wood, 2011) with a thin-plate smoothing spline for each plot (Möhl et al., 2022). The quantification of 20% seasonal growth was based on 2020 and 2021 only, as first scans occurred comparably late in 2022 [15 ± 8 (SD) days after snowmelt depending on plot; due to very early snowmelt in this year]. Leaf lengths at the day of snowmelt were set to 0.5 cm (by adding 10 datapoints of 0.5 cm per plot). Changes in total root length during different periods were calculated as the net difference between the last scan of the respective period and the last scan of the previous period. Negative values (declines) in root lengths were thus due to disappearance of roots, in all cases not accompanied by a higher fraction of dead-looking roots. Changes in root length over winter were determined by subtracting the root length of the last scan of the previous season from the first scan of the new season for each plot. Root length from the two tubes per plot was averaged prior to statistical analyses, but the two measurements correlated well (Pearson’s r = 0.87). Judged by the last scan in 2022, plot identity explained four times more of the variance in root length than tube identity within plot (P<0.001). Least absolute shrinkage regression (LASSO, package ‘glmnet’; Friedman et al., 2010) with bootstrapping was used to pinpoint plant species whose aboveground cover were related to the mean seasonal root length increment, calculated as the difference between the maximum root length of the season and its first value after snowmelt. The relationship between the species selected by LASSO and root length was further analyzed with linear mixed effects models (nlme package, Pinheiro et al., 2021).

Treatment effects on leaf lengths and root growth were analyzed using linear mixed effects models with block and plot as random effects (the latter only in the case of repeated measures). Model assumptions of residual distribution were assessed visually. *Post-hoc* contrasts between treatments were calculated using the package ‘emmeans’ (Lenth, 2021). If not stated otherwise, estimates are reported as means ± SE.

## Results

### Environmental conditions

**Figure 3:**
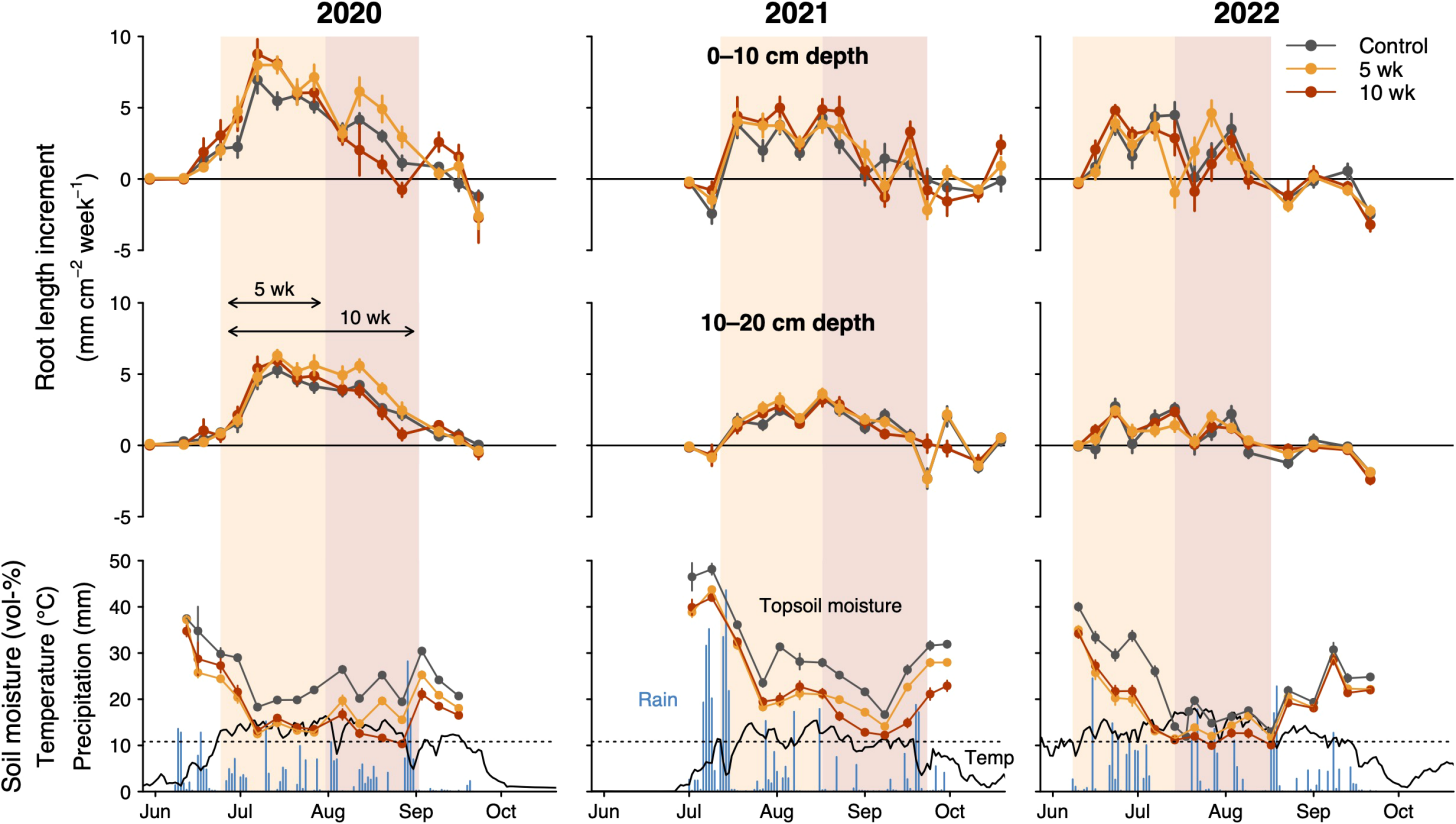
Effects of drought (5- and 10-weeks) on weekly root length increments and soil moisture over three growing seasons (2020–2022). Rhizotrons were installed in July 2019 (see data for the installation year in **Supplementary Figure S3**). Top panels show data for the upper soil layer (0–10 cm) and middle panels for the lower soil layer (10–20 cm). Lower panels present soil moisture (0–5 cm depth) in the different drought treatments, temperature in controls at 3–4 cm soil depth and on-site daily precipitation. Points indicate means and error bars ± 1 SE. Dashed lines indicate the vol-% soil moisture at which the permanent wilting point is reached.

The four growing seasons (2019–2022) differed markedly in snowmelt timing, which occurred at the end of June in 2019, early June in 2020, early July in 2021 and already at the end of May in 2022 (**Supplementary Figure S2**). The snowmelt treatment on average advanced and delayed snowmelt by 4.7 ± 0.6 and 10.8 ± 0.7 days, respectively (**Supplementary Figure S2**). Rain-out shelters excluded 85–120 mm of precipitation during the first five weeks except for 2021, when heavy rain- and snowfalls at the beginning of the treatment added up to 194 mm within the five weeks. The subsequent five weeks of the 10-weeks drought excluded another 70–115 mm of rain. This led to a significant decline in soil moisture in the topsoil (0–5 cm), dropping to values around the permanent wilting point in the droughted plots (**Table 1**; **Supplementary Figure S2**). At lower soil depths, the effect was less pronounced (**Supplementary Figure S2**) and disappeared at 35–40 cm soil depth, where soil moisture was generally lower due to higher stone content. Post-drought soil moisture recovered partly but never reached levels of controls before the end of the growing season (**Table 1**; **Figure 3**). Rain-out shelters reduced irradiance by 13% and led to slightly higher soil temperatures (0.8–1.2 K, **Table 1**). A more detailed account of the environmental conditions throughout the entire study duration is given in **Supplementary Figure S2** and Möhl et al. (2023).

**Table 1:** Mean soil temperature and minimum soil moisture (± SE; mean of 2019-2022) in controls and drought treatments during the period between snowmelt and the end of the 5-week drought treatment (0–5 wk), the period when only the 10-week drought plots were covered by rain-out shelters (5–10 wk), the following period until the last root scan of the season (3–6 weeks later), and during winter (last root scan until following snowmelt; includes temperatures > 0 °C in late autumn).

Depth	Treatment	0–5 wk	5–10 wk	Autumn	Winter
Temperature (°C)					
	Control	12.0 ± 0.2	12.6 ± 0.1	8.4 ± 0.1	0.3
	5-wk	12.5 ± 0.3	12.5 ± 0.1	8.5 ± 0.1	0.3
	10-wk	12.7 ± 0.2	13.8 ± 0.1	8.5 ± 0.1	0.3
Moisture (vol-%)					
0–5 cm	Control	21.8 ± 0.7	17.8 ± 0.5	22.1 ± 0.6	–
	5-wk	13.5 ± 0.6	14.8 ± 0.5	19.4 ± 0.5	–
	10-wk	13.9 ± 0.6	11.0 ± 0.5	16.0 ± 0.7	–
15–20 cm	Control	22.9 ± 1.0	20.4 ± 1.0	22.2 ± 0.9	–
	5-wk	18.9 ± 2.0	18.0 ± 1.8	20.3 ± 1.9	–
	10-wk	19.1 ± 1.5	15.2 ± 1.7	18.9 ± 1.6	–

Temperature was measured at 3–4 cm and soil moisture at the indicated depth (0–5 cm and 15–20 cm). SE of winter temperature was below 0.1, soil moisture was not assessed in winter.

### Seasonal dynamics of leaf expansion and root length increments

**Figure 4:**
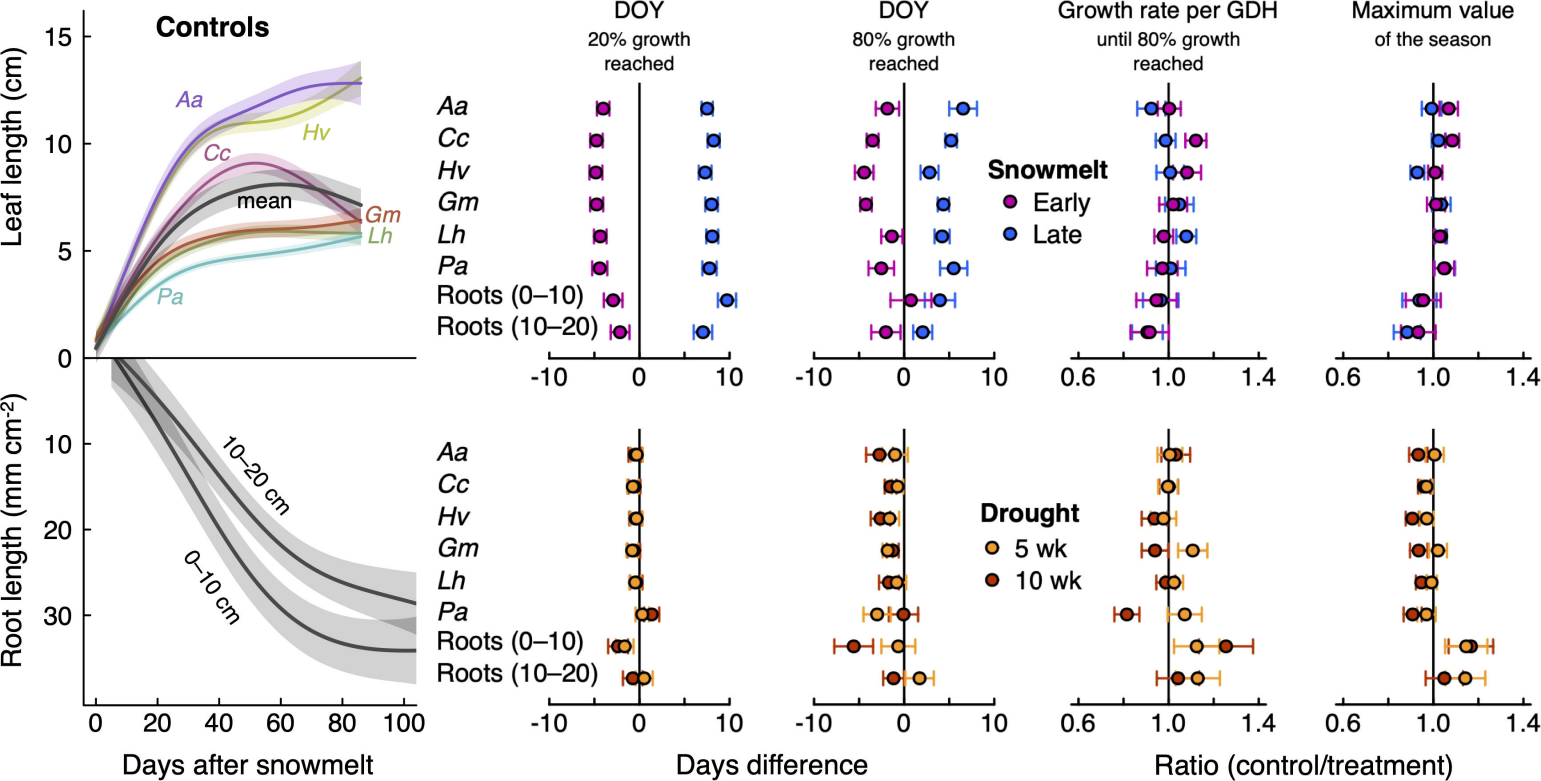
Left: Seasonal expansion of leaf (top) and root length (bottom; relative to the first root scan of the season, which was set to zero), expressed by a smoothed curve (GAM) with standard errors (shaded area). Mean leaf length across six species is shown in grey. Right: Effects of shifted snowmelt and drought on intra-seasonal growth partitioning and maximum values of leaf and root expansion. DOY, day of year; GDH, growing degree hours (> 5 °C). Data were averaged over three seasons; 2019–2021 for leaves and 2020–2022 for roots. Points and error bars depict mean ± SE of the difference to controls (black vertical zero lines). The ratios for growth rates and maximum values were obtained by dividing the control by the treatment values. Root data (total plant community) are shown for the two soil layer separately (0–10 cm, 10–20 cm). Species abbreviations (leaves): Aa, *Anthoxanthum alpinum*; Hv, *Helictotrichon versicolor*; Cc, *Carex curvula*; Gm, *Geum montanum*; Lh, *Leontodon helveticus*; Pa, *Potentilla aurea*. ANOVA results are given in **Table 2**.

Leaf expansion and root length increments started without notable delay after snowmelt (**Figure 4**). The first 20% of growth in plots without snow manipulation took about 5.0 ± 0.3 (*Geum*) to 6.8 ± 0.3 days (*Helictotrichon*) for leaves (ca. 500 and 700 GDH > 5 °C, respectively), and 23.5 ± 0.8 days for roots (3200 GDH). Roots generally grew over a much longer period than leaves (**Figure 4**), and 80% of seasonal root growth was on average reached after 57.0 ± 1.8 days (10,000 GDH), compared to a range of 20.0 ± 0.6 (*Geum*) to 26.4 ± 1.0 days (*Anthoxanthum*) for leaves (2700–4200 GDH). Maximum leaf lengths obviously differed between species but values were very consistent between years (data not shown).

**Figure 5:**
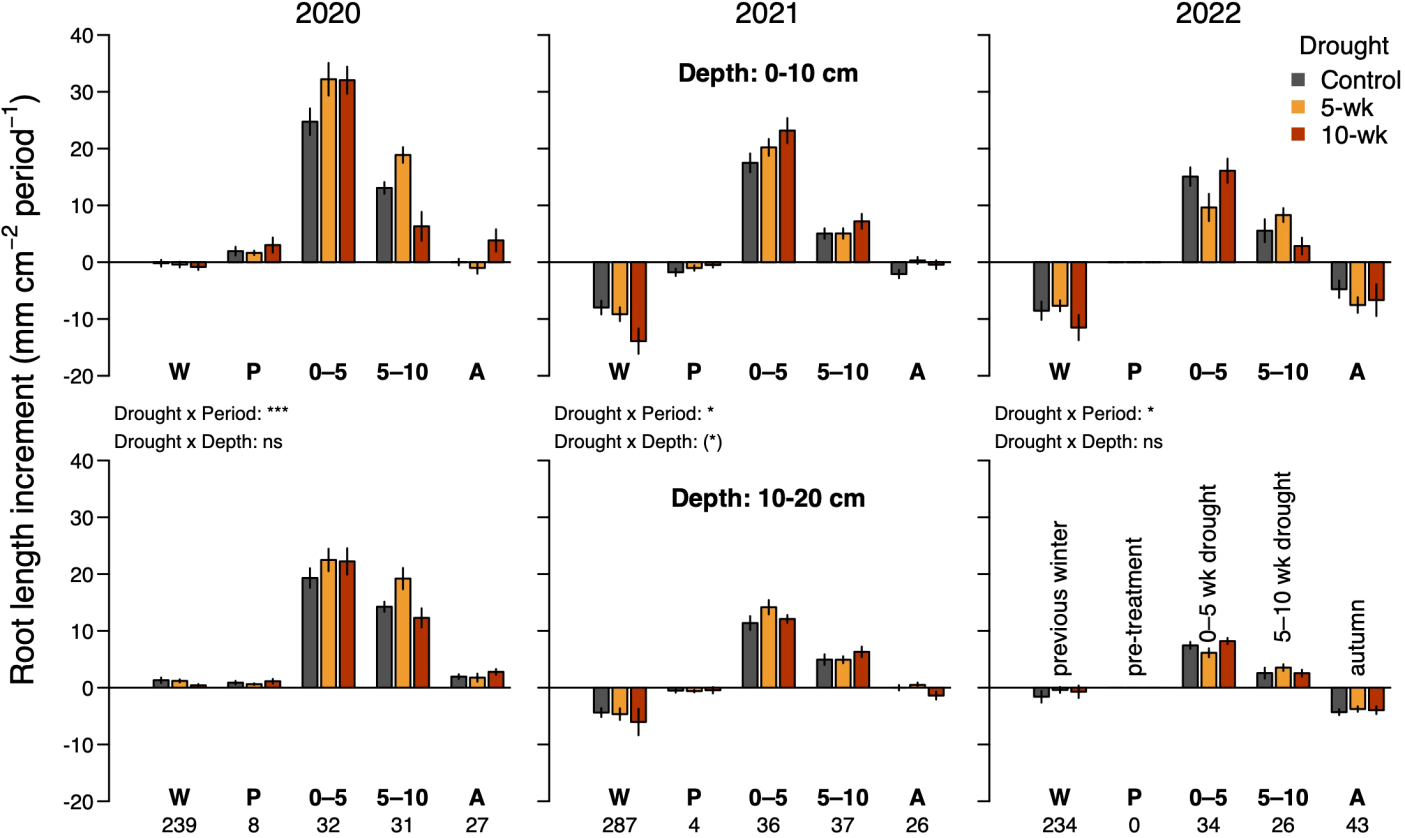
Effects of drought on the root length increment during five different seasonal periods in 2020–2022. The duration of each period is indicated below the bottom labels of the x-axis (days). Winter (W): late autumn in the previous year until snowmelt. Pre-treatment (P): Snowmelt until the start of drought. 0–5: 0 to 5 weeks of drought. 5–10: 5 to 10 weeks of drought. Autumn (A): after the 10-wk drought until snowfall. Values represent the difference between the last scan of the focal period and the last scan of the previous period. The last scan of the 5–10 period in 2022 was omitted (see Methods), reducing its duration by one week. Top panels show data for the upper soil layer (0–10 cm) and bottom panels for the lower soil layer (10–20 cm). ANOVA results for the interactions between drought and period or soil depth are indicated for each year separately (*P<0.05, ***P<0.001). Full ANOVA results are given in **Supplementary Table S1**. ns, not significant.

The increase in net root length during the growing season was comparatively low in the first year when rhizotrons were installed (18 ± 3 mm cm^-2^ on average, **Supplementary Figure S3**, excluded from analyses due to potential installation effects), peaked in the second year (42 ± 3 mm cm^-2^; **Figure 3**) and then gradually became less in the third (23 ± 3 mm cm^-2^) and fourth year (18 ± 3 mm cm^-2^), assumingly because rooting density in the soil surrounding the rhizotron tubes became increasingly saturated. Surprisingly, seasonal root length increments were not related to the fraction of bare ground nor to the plant cover of the dominant *Carex*. However, our analysis suggested that plots with more *Anthoxanthum* produced more roots (P < 0.001), which was consistent between estimates of the entire central m^2^ and the immediate surrounding of each tube (P < 0.001). Root length increments were always highest during the first 5–7 weeks of the season, lower in the following five weeks and almost negligible during the remaining autumn, which encompassed 3–6 weeks depending on year (**Figure 5**; **Supplementary Figure S4**). We even observed a decline in root length during a cold spell early October 2022, when soil temperature dropped to almost 0 °C (**Figures 3**, **5**). This general pattern of seasonal root length development was similar between soil depths but more pronounced in the upper soil layer.

In contrast to what we hypothesized (H3), total root length in the topsoil declined considerably over the last two of the three observed winters (**Figure 5**). In controls, it declined by 15 ± 3% during the 2020/2021 winter and by 7 ± 2% during the 2021/2022 winter, amounting to a substantial 24% and 28% of the previous season’s increase in root length, respectively (**Figure 5**; P < 0.001 for both years). In the deeper soil layer, a decline (by 12 ± 2%) was only observed in the second winter, corresponding to 16% of the previous season’s gain in root length (**Figure 5**; P = 0.006).

Root diameters in the rhizotron images were to 99% smaller than 1 mm and the majority (83%) even lower than 0.3 mm (**Supplementary Figure S5**). There was no significant difference in the diameter distribution between the upper and lower soil layer (**Supplementary Figure S5**) and seasonal growth dynamics were generally very similar between diameter classes of 0–0.3 mm and >0.3mm (not shown). Specific root length in ingrowth cores was 149 m g^-1^ in the upper soil layer and 20 ± 3% higher in the deeper soil layer (P = 0.015; **Supplementary Figure S6**).

### Effects of the snowmelt treatment

The snowmelt treatment had little effect on maximum leaf lengths or seasonal root length increments but caused shifts in the timing of growth (**Figure 4**). The date when 20% growth was reached shifted significantly with the timing of snowmelt: for leaves, it occurred 4.1–4.8 days earlier (depending on species; means across three years) with reduced snow cover, and 7.3–8.3 days later with raised snow cover (P < 0.001; **Table 2**). As expected (H1), this shift was paralleled by the roots, which reached 20% of the seasonal root length increment 2.5 ± 1.0 days earlier and 8.4 ± 1.0 days later when exposed to earlier or later snowmelt, respectively (**Figure 4**: P < 0.001; **Table 2**). The attainment of 80% growth still carried this snowmelt signal, albeit to a lesser degree (**Figure 4**; **Table 2**). During the period of major root proliferation, mean growth rates related to soil temperature sums (GDH > 5 °C) were similar between snowmelt treatments (P = 0.46; **Table 2**; **Figure 4**; **Supplementary Figure S4**), indicating that growth rates were mainly a function of temperature. While there was no consistent effect of snowmelt timing on total seasonal leaf or root increments, we observed a few species-specific differences between snowmelt treatments (**Figure 4**). Altered snowmelt had no effect on mean SRL (**Supplementary Figure S6**).

**Table 2:** ANOVA results for seasonal growth and maximum values of leaf (six species) and root expansion (total plant community) under altered snowmelt and drought treatments (see **Figure 4**).

Predictor variables	DOY 20% growth			DOY 80% growth			Growth rate per GHD until 80% growth			Maximum value of the season		
Leaves	χ ^2^	df	P	χ ^2^	df	P	χ ^2^	df	P	χ ^2^	df	P
Species	**268.4**	**5**	**<0.001**	**706.1**	**5**	**<0.001**	**1152.6**	**5**	**<0.001**	**3662.9**	**5**	**<0.001**
Snow	**321.4**	**2**	**<0.001**	**202.4**	**2**	**<0.001**	1.5	2	0.475	3.4	2	0.183
Drought	0.6	2	0.731	**7.6**	**2**	**0.022**	**6.7**	**2**	**0.035**	**10.0**	**2**	**0.007**
Species x Snow	**40.4**	**10**	**<0.001**	**29.2**	**10**	**<0.001**	**22.1**	**10**	**0.015**	**21.8**	**10**	**0.016**
Species x Drought	17.5	10	0.063	**21.6**	**10**	**0.017**	17.2	10	0.071	11.8	10	0.298
Snow x Drought	3.3	4	0.508	**9.8**	**4**	**0.044**	4.0	4	0.401	2.3	4	0.679
Roots												
Depth	**175.5**	**1**	**<0.001**	**17.0**	**1**	**<0.001**	**232.2**	**1**	**<0.001**	**116.6**	**1**	**<0.001**
Snow	**147.0**	**2**	**<0.001**	**6.3**	**2**	**<0.001**	1.5	2	0.462	2.2	2	0.336
Drought	2.9	2	0.231	3.1	2	0.211	2.4	2	0.297	3.6	2	0.168
Depth x Snow	**34.5**	**2**	**<0.001**	4.1	2	0.128	0.8	2	0.68	0.7	2	0.713
Depth x Drought	**6.0**	**2**	**0.049**	5.0	2	0.082	**12.0**	**2**	**0.002**	3.0	2	0.218
Snow x Drought	2.1	4	0.711	5.5	4	0.236	10.1	4	0.039	5.4	4	0.249

Data were averaged across years and analysed with mixed effect models.

P-values <0.05 are in bold.

### Drought effects

In contrast to snowmelt, drought had very little influence on the timing of leaf growth. We observed that early drought caused a slight advance (ca. 1.7 ± 0.7 days across species) of the date when 80% leaf expansion was reached (similar between 5- and 10-wk drought, P = 0.022; **Table 2**; **Figure 4**), but this effect was weak and inconsistent between species and drought duration (**Table 2**). For roots in the topsoil, drought induced a trend towards a slightly advanced date when 20% of seasonal growth was surpassed (by 1.8 ± 0.9 days, P = 0.115; significant depth x drought interaction in **Table 2**).

The long 10-wk drought significantly reduced maximum leaf length by 7.3 ± 2.1% across species (P = 0.007, **Table 2**; **Figure 4**), whereas there was no significant effect of the shorter drought. As the main leaf growth phase was surpassed, rewetting had no effect on leaf growth after either of the drought treatments. In the case of roots, we found that drought stimulated root proliferation during the first 5 weeks by 19.1 ± 7.9% (P = 0.02) in the first undisturbed season (2020) and by 19.4 ± 8.7% in the 2021-season (P = 0.03; averaged across both drought treatments), but not 2022 (**Figure 5**). Interestingly, in 2020, the growth response during the second five weeks differed markedly between the drought treatments (**Figure 3**): as soil moisture kept declining under the continuing drought of the 10-week treatment, root growth slowed down compared to controls (-22%, P=0.074, **Figure 5**), while rewetting further stimulated root proliferation by a substantial 38% and a similar effect of rewetting was observed after the 10-week treatment (+77%; P = 0.043; mean for both soil layers; **Figure 5**), although in the latter case the absolute effect was less as growth was already very low towards autumn. This stimulation during rewetting was not observed anymore in the subsequent growing seasons (2021, 2022). Against our hypothesis (H2), we found no indication that the response to drought differed strongly between soil depths (**Figure 5**; trend in 2021 only), although changes in root length were consistently less pronounced in the deeper soil layer (10–20 cm). Drought treatments affected root losses over winter in the topsoil (P = 0.058), with a 1.5 times more pronounced loss in the 10-wk drought treatment compared to the other treatments (-17 ± 2% compared to -11 ± 1%) when averaged across the winters 2020/2021 and 2021/2022 (**Figure 5**; P = 0.011; no difference between 5-wk drought and controls). Drought reduced SRL by 8.4 ± 3.6% across soil depths (P = 0.02; no difference between 5- and 10-wk treatment; **Supplementary Figure S6**) without affecting average root diameters (P = 0.54; not shown) and root diameter distribution (**Supplementary Figure S5**).

## Discussion

The short- and long-term dynamics of root growth in alpine plants are largely unknown, and even less is known about their response to a changing environment. By analyzing a large array of root images we have unearthed new insights into the *in situ* dynamics of root growth in an alpine grassland, related it to leaf expansion of different species and assessed the impact of projected climate change on both. Our results show that roots, like leaves, expand predominantly early in the season although the period of major root expansion is about twice as long as the one required to form the aboveground biomass. As expected, snowmelt led to shifts in the onset of growth but we found that it had negligible effects on total seasonal growth of leaves and roots. We highlight that drought duration greatly matters and that both, the dry period and the subsequent rewetting are relevant for the response of roots to drought events. However, rewetting is only effective when it materializes not too late in the growing season. Interestingly, there was no increase in total root length over winter but instead a pronounced decline.

### Growth dynamics within the growing season

Leaves required less than half of the heat sums of roots to reach 80% of their seasonal expansion, demonstrating that growth continues mainly belowground after the first few weeks of the season in this ecosystem. This is similar to reports for arctic tundra and boreal ecosystems (Abramoff and Finzi, 2015; Blume-Werry et al., 2016; Gallois et al., 2025), where root growth extended leaf growth, but also started later (and did not track earlier snowmelt; Darrouzet-Nardi et al., 2019). In contrast, we observed no notable delay in root compared to leaf growth at the beginning of the season, which matches our previous findings from excavated monoliths with more frequent early-season measurements (Möhl et al., 2022). The reason for this is probably that the soils in this alpine grassland hardly ever freeze (due to insulation by the snowpack) and warm up quickly after snowmelt, while arctic soils often freeze in winter and warm slowly in spring (Ives and Barry, 1974; Körner, 2021).

The substantial increase in root length we observed during the early part of each season was extremely reduced after the first two months, despite suitable growing temperatures. This finding is consistent with the hypothesis that roots are strongly dependent on recent photosynthates for growth (Abramoff and Finzi, 2015; Edwards et al., 2004; Pregitzer et al., 2000). Given that leaf senescence starts relatively early in this vegetation type (Möhl et al., 2022)—several weeks before the end of the meteorological growing season—we speculate that the senescing plant canopy does not provide enough assimilates to fuel substantial root growth in late summer. If roots rely on fresh assimilates from leaves, the early cessation of root growth could be a direct consequence of early leaf senescence, which in turn is an adaptation to cope with the increasing frost risk in late summer and autumn (Körner, 2021).

Even four years after the establishment of the rhizotrons, root diameters were rarely wider than 1 mm. This underpins that the widely used thresholds of 1–2 mm to differentiate between absorptive and transport roots (Silver and Miya, 2001) should not be applied to alpine grasslands (McCormack et al., 2015). Given that > 80% of the roots in our rhizotron images had an extremely small diameter below 0.3 mm (used previously as a threshold for absorptive roots in alpine grassland; Song et al., 2024), we expect that the majority of produced roots was absorptive.

### Links between snowmelt and growth timing

Roots and leaves responded to earlier and later snowmelt with proportional shifts in growth onset, confirming *in situ* that above- and belowground growth of this alpine grassland starts opportunistically (Möhl et al., 2022). As shoot and root growth begins shortly after snowmelt, future mismatches between growth and nutrient availability—which peaks during and after snowmelt (Bilbrough et al., 2018; Broadbent et al., 2021)—are unlikely to occur in spring, except for years when early snowmelt is accompanied by cold spells with cycles of soil freezing and thawing (Broadbent et al., 2024). Clearly, alpine plants are designed for early season activity, and missing the early nutrient pulse in exceptional years may put them at competitive disadvantage in environments where growth is often restricted by nutrient availability (Körner, 2021).

Changes in snowmelt timing hardly affected total seasonal growth in terms of maximum leaf and root lengths. In line with this, earlier results indicated no effect of altered snowmelt timing on seasonal plant productivity assessed by biomass harvests (Möhl et al., 2023). Our data revealed that growth rates were very similar among treatments when expressed per growing degree hour (GDH). In an *in situ* survey of the growth dynamics of the dominant *Carex curvula* across different snowmelt regimes, we found that growth rates after snowmelt were strongly modulated by temperature and patches with later snowmelt made up for the ‘lost time’ through higher growth rates (Möhl et al., 2022). The effect of earlier snowmelt on growth dynamics is thus considered to be relatively weak when early snowmelt is followed by cold weather. More significant advances of growth with earlier snowmelt may occur as spring temperatures keep rising in the future (Zehnder et al., 2025). This may leave plants more exposed to frost damage and disrupt nutrient cycling through mismatches between plant and soil microbial activity (Broadbent et al., 2024). Similarly, such mismatches due to early snowmelt could result from extended periods of low plant activity in late summer when soil temperatures are still high (Möhl et al., 2022).

### Growth dynamics during drought and recovery

We previously reported that only the shorter drought treatment stimulated root production in ingrowth cores in this grassland (Möhl et al., 2023). The rhizotron data presented here offer an explanation for this finding, as they show clear differences in the response of root growth to the two drought treatments in the first (undisturbed) growing season (which was the fourth season of the longer-term experiment with recurrent drought treatment). Specifically, we found that moderate drought conditions as well as post-drought rewetting both stimulated root growth, while ongoing drought (>5 weeks) impaired it. In most previous studies it remained unidentified whether stimulated root production in response to drought resulted from the drought period itself or from the post-drought rewetting phase. Both are potential drivers of root growth; the former as it triggers plant responses to invest into water and nutrient uptake and the latter because rewetting is often accompanied by nutrient pulses due to rapid mineralization of dead microbes or soil organic matter (Schimel, 2018), providing the substrate to fuel compensatory growth (Schärer et al., 2025; van Sundert et al., 2020). Our results thus emphasize that the duration of drought is a crucial aspect of root responses (Guasconi et al., 2023).

The fact that we mainly observed this effect in one (2020) of three growing seasons could stem from the differences in environmental conditions between years. For example, the effect of rewetting on soil moisture was less pronounced in 2021 and 2022 compared to 2020 due to varying precipitation patterns. The absence of a rewetting effect in later years may also be accountable to the increasingly saturated rooting density, leaving less room for additional root growth. In undisturbed soil with a higher abundance of old roots, even if it is densely rooted, dying roots would periodically open up spots for fresh root colonization. In our case, embedding the tubes in a thin layer of sieved soil meant that all roots were at maximum three years old at the end of this study and it might thus take several years longer before root mortality becomes frequent.

Contrary to our hypothesis, root responses to drought were largely independent of soil depth, suggesting that drought does not shift root growth to deeper soil layers in this ecosystem. Similar observations come from temperate lowland and sub-alpine grasslands (Prechsl et al., 2015) and may be a result of aggravating nutrient limitation under drought rather than low water availability *per se*. Root growth in deeper soil layers would rather improve water acquisition than nutrient foraging, as nutrient cycling mainly occurs in the topsoil. We found that roots from droughted ingrowth cores had lower SRL, which is a common response to drought in grassland (de Vries et al., 2016; Zhou et al., 2018) and may improve hydraulic safety and root lifespan (due to changes in root tissue density; Freschet et al., 2021b), although in our case the longer drought was associated with higher mortality over winter despite lower SRL.

### No winter root growth

Despite that roots of several common alpine grassland species are capable of growing at temperatures close to 0 °C (albeit with strongly impaired root branching and cell differentiation; Nagelmüller et al., 2017), we observed no increase in root length over winter. The lack of root growth may be due to the absence of aboveground demand for nutrients and water (no sink activity) or the paucity of fresh photo-assimilates to fuel root growth during winter. While rhizomes and roots of alpine plants are packed with large concentrations of carbohydrates (Tolsma et al., 2007) that could potentially be reallocated to growth in the absence of photosynthetically active tissues (Hiltbrunner et al., 2021; Körner et al., 2019), the alpine grassland studied here apparently does not use these reserves for winter root growth.

Rather than an increase, we found a marked loss of root length in the last two of the three winters studied, especially in the upper soil layer, indicating net mortality of roots in winter, which accounts for more than two-thirds of the year in temperate alpine ecosystems. Similar to our findings, a study with montane grassland on the Tibetan Plateau evidenced a massive decline in standing root length during winter, dropping to 50% of peak summer values (Wu et al., 2021). Hence, alpine grasslands contrast ecosystems at lower elevation with milder winters that allow continued root growth (Radville et al., 2016). Root meristems are often not dormant like aboveground meristems, but instead continuously capable of growth—and therefore also more susceptible to frost damage (Tierney et al., 2003). It is not known whether roots of alpine plants enter environmentally triggered endodormancy, but the finding that the autumnal cessation of root growth of two arctic graminoids is related to the shortening photoperiod at least points in this direction (Shaver and Billings, 1977).

Interestingly, winter losses were most pronounced in the long drought treatment, providing evidence that changed life conditions and disturbances during summer can affect ecosystem processes occurring in winter. This finding may have various causes that await testing. It is conceivable that interactions with soil microbes play a role, as drought often entails changes in microbial abundance and community composition (Canarini et al., 2021; de Vries et al., 2018; Fuchslueger et al., 2014), which could have consequences for decomposition rates (Glassman et al., 2018). Alternatively, increased winter losses could be a direct consequence of the treatment, for example if intense drought led to increased mortality of roots that then get decomposed during winter. However, we visually observed almost no dead roots in our study, which is similar to findings from arctic heath (Balogianni et al., 2016). Either the young roots growing along our rhizotrons had (i) a low mortality rate, or (ii) dying finest roots (diameters < 0.3mm) decomposed readily over winter. The latter is supported by a study in alpine grassland of the Tibetan Plateau, which found that the mortality rate increases with decreasing root diameter (Wang et al., 2016). Otherwise, declines in root length resulted from (iii) belowground herbivory (Hunter, 2001), which was not directly observable by our method. Novel machine learning models designed to differentiate between existing and newly formed roots in temporal sequences offer a promising way to assess root turnover (Gillert et al., 2023), but await an application for root-dense alpine grassland as the one studied here.

## Conclusions

Root development and growth are fundamental aspects of ecosystem functioning, as roots globally account for 22–67% of total biomass (Ma et al., 2021). And in this respect, alpine ecosystems are an extreme case, as up to 90% of their biomass lays belowground, yet little is known about the seasonal dynamics of root growth in these ecosystems. We here provide *in situ* evidence that the roots of the most common alpine grassland type in the European Alps start growing quickly after snowmelt and that the major period of root growth occurs in the first 1–2 months after snowmelt compared to 3–4 weeks for leaves. Extreme events therefore have the potential to affect growth very differently above- than belowground, depending on their timing. That the stimulation of root growth we observed under drought and rewetting was transient emphasizes the need for experiments lasting several years, with more consistent effects to be expected once the experimental duration covers the average root lifespan of the different plant species (which is often unknown). The fact that the majority (> 80%) of roots were less than 0.3 mm in diameter and that there were no discernible effects of the experimental treatments on the root diameter distributions, even across the study years, makes the assessment of root longevity an important focus for the future—especially as roots contribute significantly to the carbon cycle in these ecosystems (besides the provision of other fundamental ecosystem services), simply due to their enormous mass and high carbon concentrations. Finally, our finding that roots do not grow, but rather decline, during the long, snow-covered winter suggests that roots enter a dormant phase during winter. Remarkably, higher root losses following the severe drought treatment hint towards unexplored legacy effects of drought on the longevity or decomposition of roots.

## Data Availability

The datasets presented in this study can be found in online repositories. The names of the repository/repositories and accession number(s) can be found below: Figshare repository: https://doi.org/10.6084/m9.figshare.22141037.
