# Supplementary material for: Recurrent summer drought temporarily stimulates fine root growth but enhances winter root losses in alpine grassland
Source: Front Plant Sci. 2025 Jul 30;16:1625076. doi: 10.3389/fpls.2025.1625076 (PMC12344270; doi:10.3389/fpls.2025.1625076)
Supplement: Supplementary file 1 [file SupplementaryFile1.pdf]

Supplementary Material

Table S1: ANOVA results for the effects of the drought treatments on root growth rates at different soil depths and at different periods (see Fig. 5). Data was analysed with mixed effect models for all three years together (left; 2020–2022) and for each year separately (right). Variables with P-values <0.05 are in bold

**All years**	χ 2	df	P
**Year**	345.5	2	**<0.001**
**Period**	2295.1	4	**<0.001**
Depth	1.8	1	0.174
Drought	1.1	2	0.5776
**Year x Period**	561.0	8	**<0.001**
**Year x Depth**	12.8	2	**0.002**
Year x Drought	1.2	4	0.585
**Period x Depth**	156.6	4	**<0.001**
**Period x Drought**	20.8	8	**0.008**
Depth x Drought	0.5	2	0.741

**Year 2020**	χ 2	df	P
**Period**	1127.2	4	**<0.001**
Depth	2.8	1	0.097
Drought	0.1	2	0.966
**Period x Depth**	43.5	4	**<0.001**
**Period x Drought**	44.6	8	**<0.001**
Depth x Drought	1.4	2	0.505

**Year 2021**	χ 2	df	P
**Period**	1099.0	4	**<0.001**
Depth	2.1	1	0.151
Drought	2.5	2	0.286
**Period x Depth**	54.6	4	**<0.001**
**Period x Drought**	18.0	8	**0.021**
Depth x Drought	5.6	2	0.061

**Year 2022**	χ 2	df	P
**Period**	619.2	3	**<0.001**
Depth	1.1	1	0.306
Drought	0.3	2	0.864
**Period x Depth**	86.9	3	**<0.001**
**Period x Drought**	12.7	6	**0.048**
Depth x Drought	2.5	2	0.29

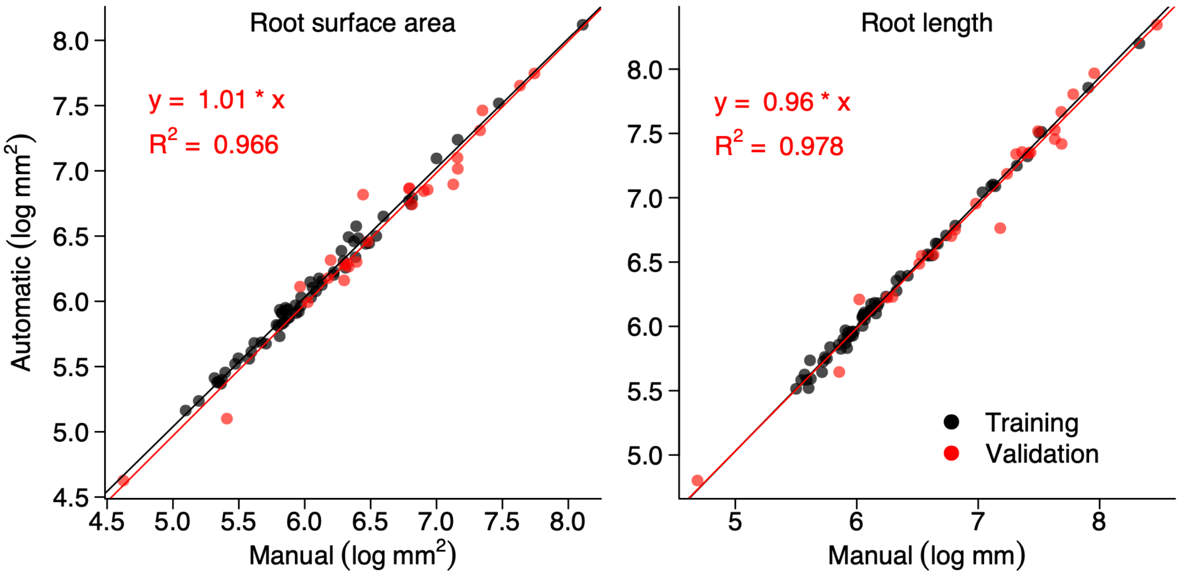

Figure S1: Comparison between estimated and manually measured root surface area and root length in the training (61 images) and validation dataset (25 images). Training images from another project are not included here. Binary images (roots white, background black) were either created manually using rhizoTrak (v 1.3) or automatically using the trained neural network. Root surface area and root length were estimated for manually and automatically segmented images using the software RhizoVision Explorer (v. 2.0.3).

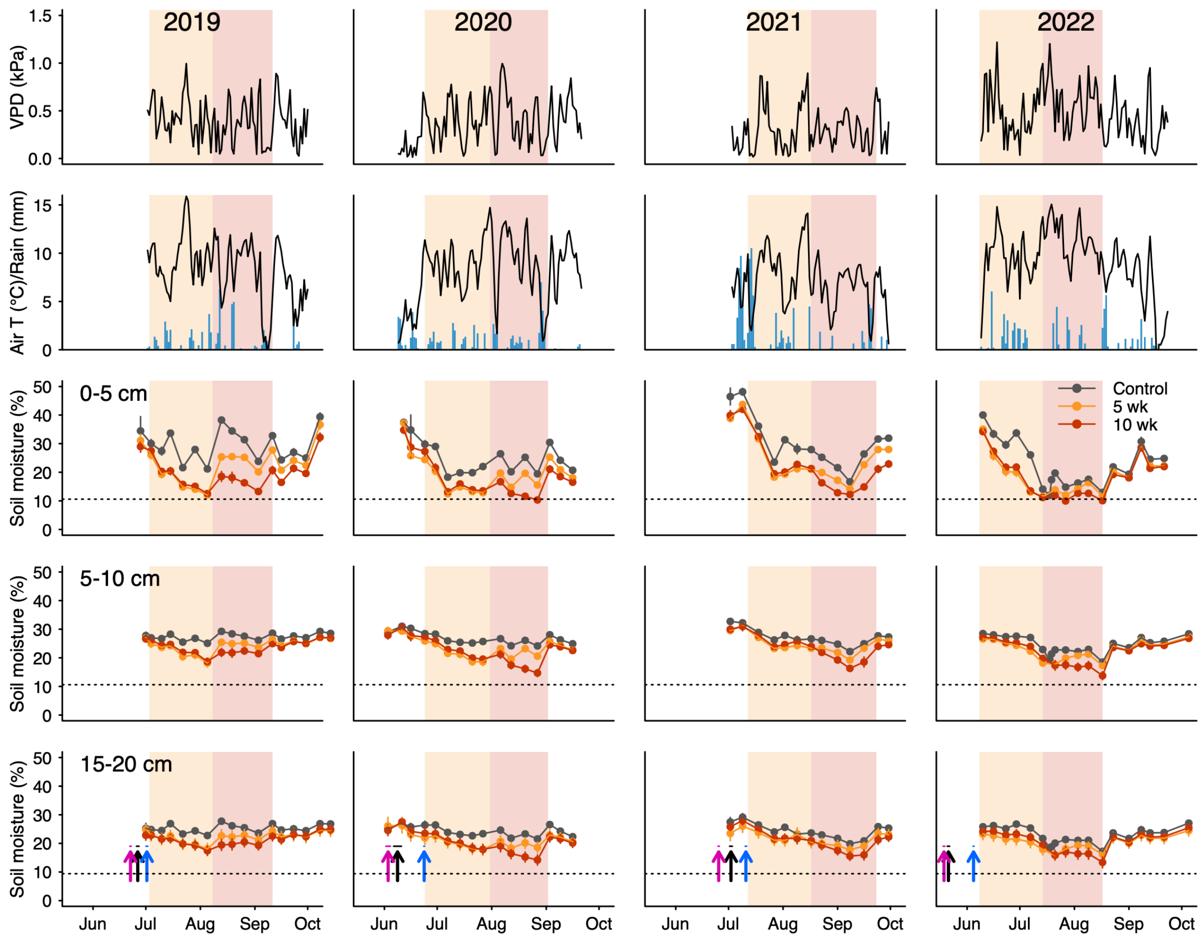

Figure S2: Environmental conditions at the study site. From top to bottom: Vapour Pressure Deficit (VPD, calculated from relative humidity and air temperature (using the R-package 'plantecophys', Duursma 2015), air temperature (1.5 m above the ground), daily precipitation, volumetric soil moisture in the topsoil (0–5 cm, Theta probe), at 5–10 cm and 15–20 cm soil depth (Profile probe, mean ± SE). Mean snowmelt dates are indicated with arrows in the bottom panels (black: controls; pink: snow removal; blue: snow addition; horizontal lines on top of the arrows show ± SE). Dotted horizontal lines indicate the site-specific permanent wilting point.

Duursma RA. 2015. Plantecophys - An R Package for Analysing and Modelling Leaf Gas Exchange Data. PLoS ONE **10(11)**: e0143346. DOI: 10.1371/journal.pone.0143346

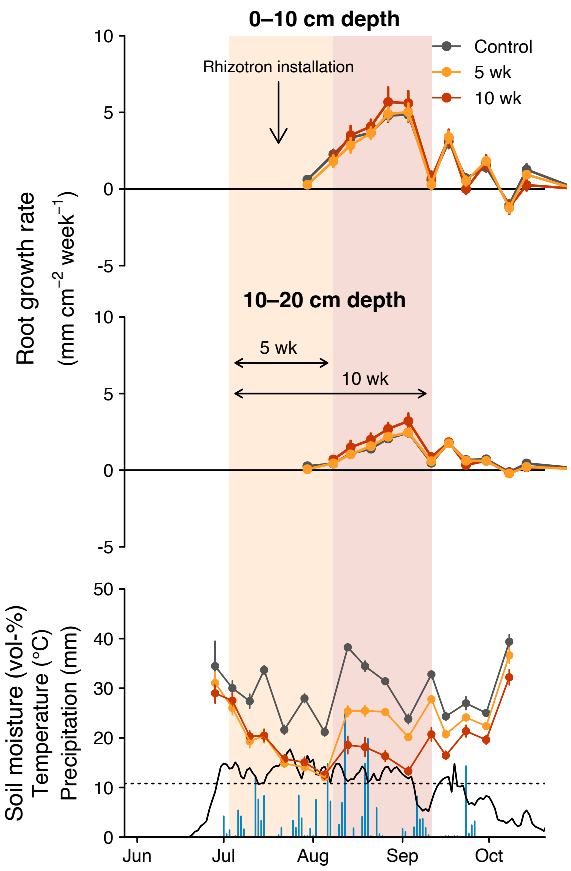

Figure S3: Effects of drought on the weekly root growth rates in 2019, when rhizotron tubes were installed. Top panel shows data for the upper soil layer (0–10 cm) and middle panel for the lower soil layer (10–20 cm). Lower panel shows soil moisture (0–5 cm depth) in the different drought treatments, soil temperature in controls and on-site precipitation. Points indicate means and error bars ± 1 SE. Dashed line indicates the vol-% soil moisture (at 10 cm) reaching the permanent wilting point.

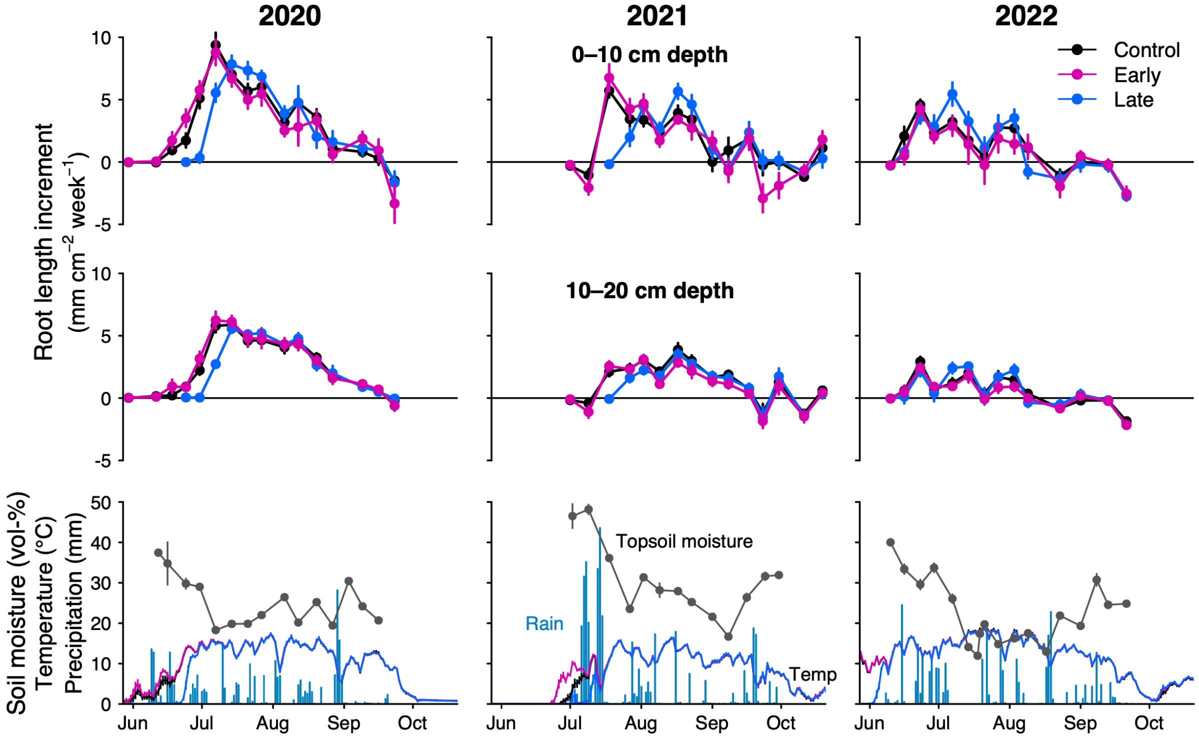

Figure S4: Effects of the snowmelt treatments on weekly root length increments over three growing seasons (2020–2022). Top panels show data for the upper soil layer (0–10 cm) and middle panels for the lower soil layer (10–20 cm). Lower panels present soil temperature (3–4 cm soil depth) in the different snowmelt treatments, soil moisture (0–5 cm depth) in controls and on-site daily precipitation. Points indicate means and error bars ± 1 SE.

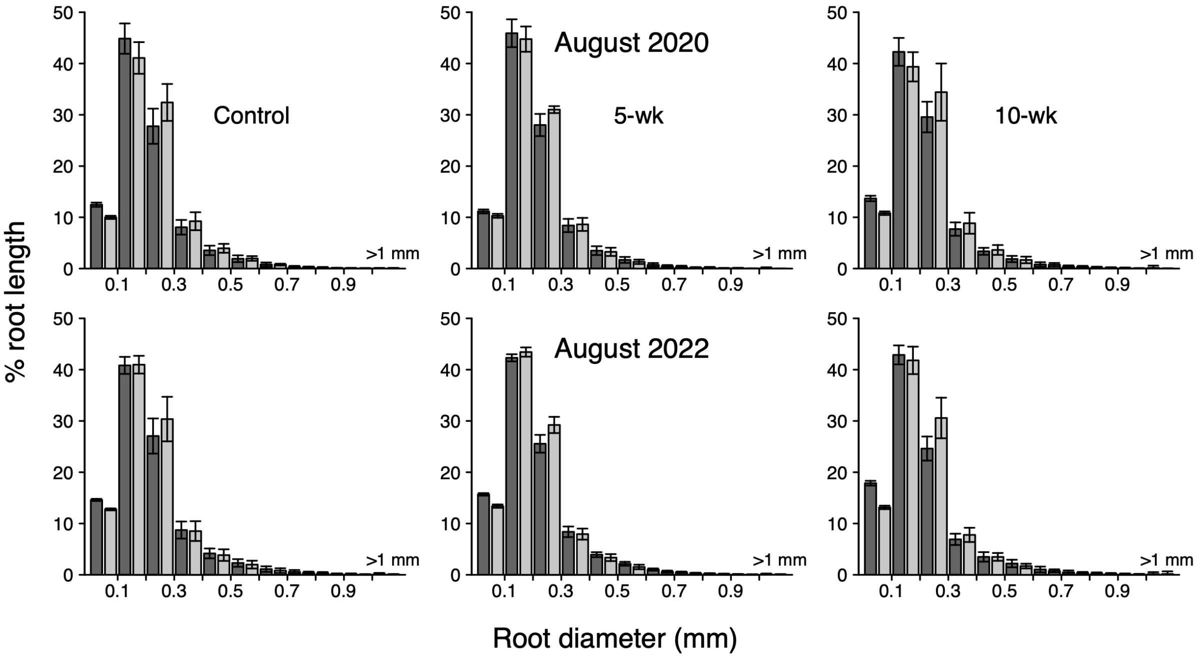

Figure S5: Root length distribution (%) across diameters in control plots in August 2020 (upper row of panels, one year after installation) and 2022 (lower panels), separated by drought treatment (left to right). Bar colours indicate 0–10 cm depth (dark grey) and 10–20 cm soil depth (light grey).

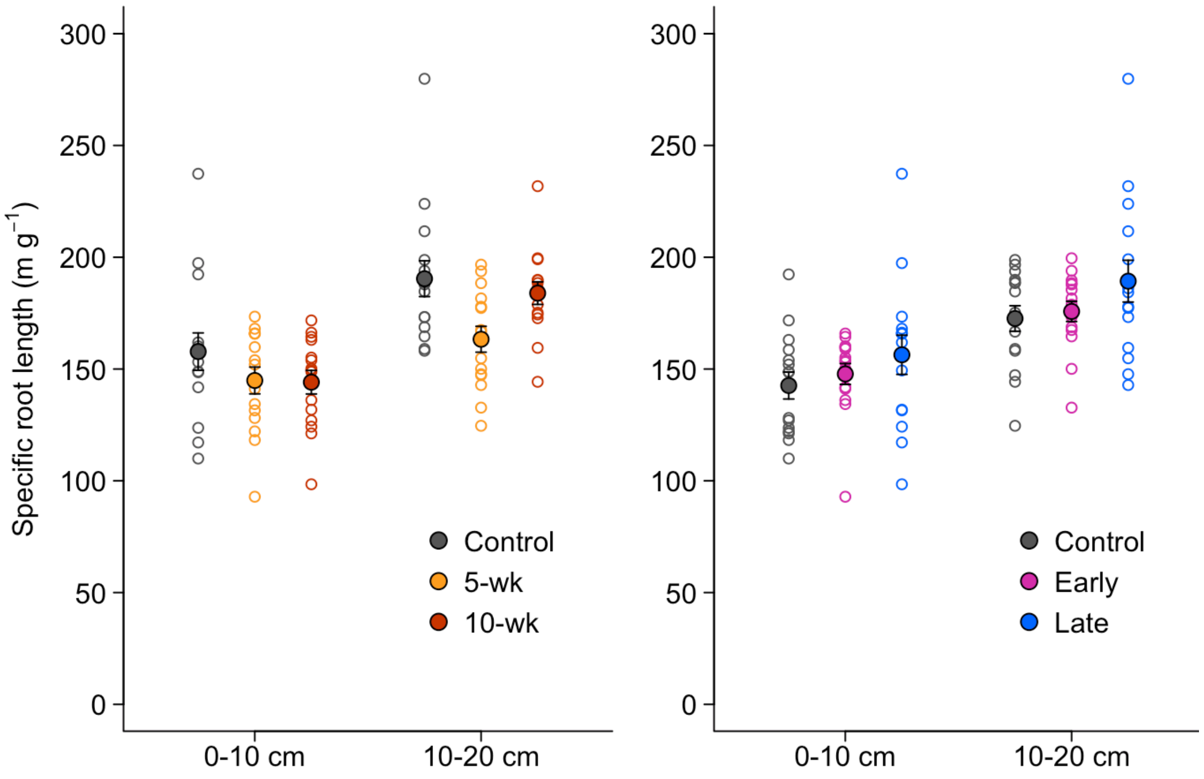

Figure S6: Specific roots length (SRL) in roots from in-growth cores indicate lower SRL under drought and higher SRL in the deeper soil layer.
